# Ultrasound prediction of Zika virus-associated congenital injury using the profile of fetal growth

**DOI:** 10.1371/journal.pone.0233023

**Published:** 2020-05-13

**Authors:** Christie L. Walker, Noah Ehinger, Brittney Mason, Elizabeth Oler, Marie-Térèse E. Little, Eric O. Ohuma, Aris T. Papageorghiou, Unzila Nayeri, Christine Curry, Kristina M. Adams Waldorf

**Affiliations:** 1 Division of Maternal-Fetal Medicine, Department of Obstetrics & Gynecology, University of Washington, Seattle, Washington, United States of America; 2 Department of Obstetrics & Gynecology, University of Miami Health System, Miami, Florida, United States of America; 3 4^th^ Dimension Biomedical Research Communications, Victoria, British Columbia, Canada; 4 Nuffield Department of Medicine, Centre for Tropical Medicine and Global Health, University of Oxford, Oxford, United Kingdom; 5 Nuffield Department of Orthopaedics, Rheumatology & Musculoskeletal Sciences, Centre for Statistics in Medicine, University of Oxford, Oxford, United Kingdom; 6 Nuffield Department of Obstetrics & Gynaecology and Oxford Maternal & Perinatal Health Institute, Green Templeton College, University of Oxford, Oxford, United Kingdom; 7 Division of Maternal-Fetal Medicine, Department of Obstetrics & Gynecology, University of Miami Health System, Miami, Florida, United States of America; 8 Department of Obstetrics & Gynecology, University of Washington, Seattle, Washington, United States of America; 9 Department of Global Health, University of Washington, Seattle, Washington, United States of America; 10 Sahlgrenska Academy, University of Gothenburg, Gothenburg University, Gothenburg, Sweden; Johns Hopkins University, UNITED STATES

## Abstract

Zika virus (ZIKV) is a mosquito-transmitted flavivirus, recently linked to microcephaly and central nervous system anomalies following infection in pregnancy. Striking findings of disproportionate growth with a smaller than expected head relative to body length have been observed more commonly among fetuses with exposure to ZIKV in utero compared to pregnancies without ZIKV infection regardless of other signs of congenital infection including microcephaly. This study’s objective was to determine the diagnostic accuracy of femur-sparing profile of intrauterine growth restriction for the identification of ZIKV-associated congenital injuries on postnatal testing. A retrospective cohort study of pregnant women with possible or confirmed ZIKV infection between January 1, 2016 and December 31, 2017 were included. Subjects were excluded if no prenatal ultrasound was available. A femur-sparing profile of growth restriction determined using INTERGROWTH-21^st^ sonographic standard for head circumference to femur length (HC: FL). Congenital injuries were determined postnatally by imaging, comprehensive eye exam and standard newborn hearing screen. A total of 111 pregnant women diagnosed with ZIKV infection underwent fetal ultrasound and 95 neonates had complete postnatal evaluation. Prenatal microcephaly was detected in 5% of fetuses (6/111). Postnatal testing detected ZIKV-associated congenital injuries in 25% of neonates (24/95). A HC: FL Z-score ≤ -1.3 had a 52% specificity (95% CI 41–63%), 82% negative predictive value (NPV, 95% CI 73–88%) for the detection of ZIKV-associated congenital injuries in the neonatal period. A more stringent threshold with a Z-score ≤ -2 was associated with a 90% specificity (95% CI 81–95%), 81% NPV (95% CI 77–85%). Excluding cases of fetal microcephaly, HC: FL (Z-score ≤ -2) demonstrated a similar specificity (89%, 95% CI 81–95%) with superior NPV (87%, 95% CI 84–90%). The sonographic recognition of a normally proportioned fetus may be useful prenatally to exclude a wider spectrum of ZIKV-associated congenital injuries detected postnatally.

## Introduction

A recent epidemic of Zika virus (ZIKV), a mosquito-transmitted flavivirus, has been linked to microcephaly and other fetal central nervous system anomalies following infection in pregnancy. [1, 2] Microcephaly or structural brain anomalies are diagnosed prenatally in only 4–9% of pregnancies with a ZIKV infection, but approximately 30% of infants are diagnosed with a wider spectrum of anomalies postnatally after eye examination, auditory testing and additional brain imaging. [3–7] Similar to other teratogenic viruses, infection in the first trimester is associated with the highest risk of microcephaly and the other structural and developmental anomalies. [8–10] Prenatal recognition of the congenital Zika syndrome has been limited to obvious structural anomalies such as calcifications, ventriculomegaly or microcephaly detectable by ultrasound. [2, 11–13] This strategy fails to identify all infants who are diagnosed with ZIKV-associated injury postnatally through vision and hearing tests and additional brain imaging. [7, 14] Although ultrasound remains the most available method for prenatal diagnosis of ZIKV-associated birth defects, studies suggest it is insufficient to predict the widening array of congenital ZIKV injury. [15–18] The lack of a prenatal diagnostic test to either predict or rule out ZIKV-associated anomalies represents a major clinical challenge in ZIKV-endemic areas.

Intrauterine growth restriction (IUGR) is a known sequela of ZIKV and other teratogenic viral infections [19], but whether aberrant fetal growth might predict congenital injuries in pregnancies exposed to ZIKV is unknown. We first observed an unusual femur-sparing pattern of IUGR in a nonhuman primate model of congenital ZIKV infection; in these experiments, there was an arrest of fetal head growth, while the long bones (e.g. femur) continued to grow normally. [20, 21] This striking profile of fetal growth was also seen in a majority of pregnant women from New York City acquiring ZIKV through travel; a lack of postnatal data precluded correlation with congenital ZIKV injury in this study. [22] Investigators reporting outcomes of Brazilian infants with congenital Zika syndrome have noted a similar discrepancy between fetal or neonatal length and head circumference (HC), which has been referred to as “disproportionate microcephaly”. [12, 23] Notably, this “femur-sparing” profile of IUGR contrasts with the pattern of growth restriction due to placental insufficiency (“brain-sparing IUGR”) and other viral infections (e.g. symmetric IUGR for cytomegalovirus), but is consistent with the known ZIKV tropism for fetal neural progenitor cells.

In this study, prenatal ultrasound biometric measurements of the fetus from pregnancies with a ZIKV infection were correlated with postnatal testing to determine the diagnostic accuracy of a femur-sparing profile of IUGR to predict ZIKV-associated congenital injury. We hypothesized that sonographic detection of an abnormally proportioned fetal body, defined by a small HC relative to femur length (FL), would identify a larger proportion of fetuses diagnosed postnatally with ZIKV-associated injuries. The prenatal diagnosis of aberrant fetal growth may aid in identification of neonates at high risk for impaired development who warrant early interventions.

## Material and methods

### Study design and institutional review board approval

Pregnant women were screened for possible ZIKV exposure between January 1, 2016 and December 31, 2017 at a tertiary care center in Miami, Florida. Following a positive result on screening questionnaire, women were tested according to the U.S. Centers for Disease Control and Prevention (CDC) guidelines. All pregnant women testing positive for confirmed or probable ZIKV infection between 2016 through 2017 were included in the study and stratified by gestational age at presumed exposure. When possible, the timing of exposure was based on known travel dates with gestational age determined by American College of Obstetricians and Gynecologists guidelines. Typically, prenatal ultrasound imaging was performed every 3–4 weeks per CDC recommendations.

The University of Miami investigators obtained Institutional Review Board approval (Jackson Health System, #201660689) as a retrospective chart review and informed consent was not required. Subjects were excluded if they did not meet CDC criteria for a “possible ZIKV infection” or did not have an ultrasound scan during pregnancy. Charts were abstracted and de-identified data was sent to the University of Washington for analysis of the following data variables: exposure trimester, gestational age at each ultrasound, fetal biometric measures from each ultrasound [HC, abdominal circumference (AC), FL, estimated fetal weight, ventricle and cerebellar measurements], birth defects, gestational age at intake and delivery, results of any abnormal ultrasound imaging from fetal or neonatal ultrasound, magnetic resonance imaging (MRI) or audiology tests, and Zika virus symptoms and laboratory testing results from mother and neonates, birthweight, and pregnancy outcome (termination of pregnancy, preterm birth, stillbirth, term birth). The University of Washington Institutional Review Board deemed that analysis of these variables did not engage in human subjects activity. Note that a prior publication reports the pregnancy outcomes from 88 of the 111 subjects in this study [24]; there is no scientific overlap as this study did not analyze the diagnostic accuracy of prenatal ultrasound to predict pregnancy outcomes.

### Diagnosis of Zika virus infection

Pregnant women were diagnosed with ZIKV infection according to the most current CDC guidelines at the time of testing. Women were included if laboratory testing supported a confirmed or possible ZIKV infection by either detection of: 1) ZIKV viral ribonucleic acid (RNA) in a maternal, placental or fetal specimen, 2) a positive/equivocal ZIKV IgM and ZIKV plaque reduction neutralization test (PRNT) titer ≥ 10, regardless of dengue virus PRNT value; or, 3) a negative ZIKV IgM, and positive/equivocal dengue virus IgM, and ZIKV PRNT titer ≥ 10, regardless of dengue virus PRNT value. [25] Newborns were tested according to CDC guidelines for possible congenital ZIKV infection. [26] A neonatal ZIKV infection was diagnosed by positive polymerase chain reaction (PCR) for ZIKV from serum, urine or cerebrospinal fluid.

### Ultrasound methodology

Choice of sonographic standard is critical for determining normal and aberrant fetal growth and, ideally, is validated within specific populations. Pregnant women in the continental U.S. are ethnically diverse and while U.S. ethnic-specific growth charts for fetal biometric measures exist, standards for fetal body ratios have not been published. [27] Therefore, we applied the International Fetal and Newborn Growth Consortium for the 21^st^ Century (INTERGROWTH-21^st^) standards, which was a multi-ethnic study of more than 13,000 pregnancies in 18 countries with which we could calculate HC: FL ratio Z-scores. [28, 29]

Biparietal diameter (BPD) is a fetal biometric measure used to estimate fetal weight and growth. Consistent with many centers in the United States using the Hadlock sonographic standard [30], the BPD was measured from outer to inner skull diameter; however, the INTERGROWTH-21^st^ standard requires BPD to be measured from outer to outer skull diameter. Due to these differences, we used HC rather than BPD measurements in this study.

Definitions for IUGR and microcephaly vary and different standards have been applied to congenital ZIKV infection. We reviewed the available definitions and chose typical standards of an AC Z-score ≤ -1·3 (10^th^ percentile) for IUGR and a fetal HC Z-score ≤ -2 (~2·3 percentile) for microcephaly. [31, 32] Estimated fetal weight is often used to define IUGR; however, INTERGROWTH-21^st^ fetal weight Z-scores could not be calculated due to differences in BPD measurements, as described above.

Fetal body ratios comparing HC or AC to FL were calculated using a Z-score ≤ -1·3 as a diagnostic threshold for abnormal growth trajectory, which has previously been shown to identify higher rates of growth restriction than use of a single fetal biometry. [22]

### Postnatal evaluation

**Fig 1:**
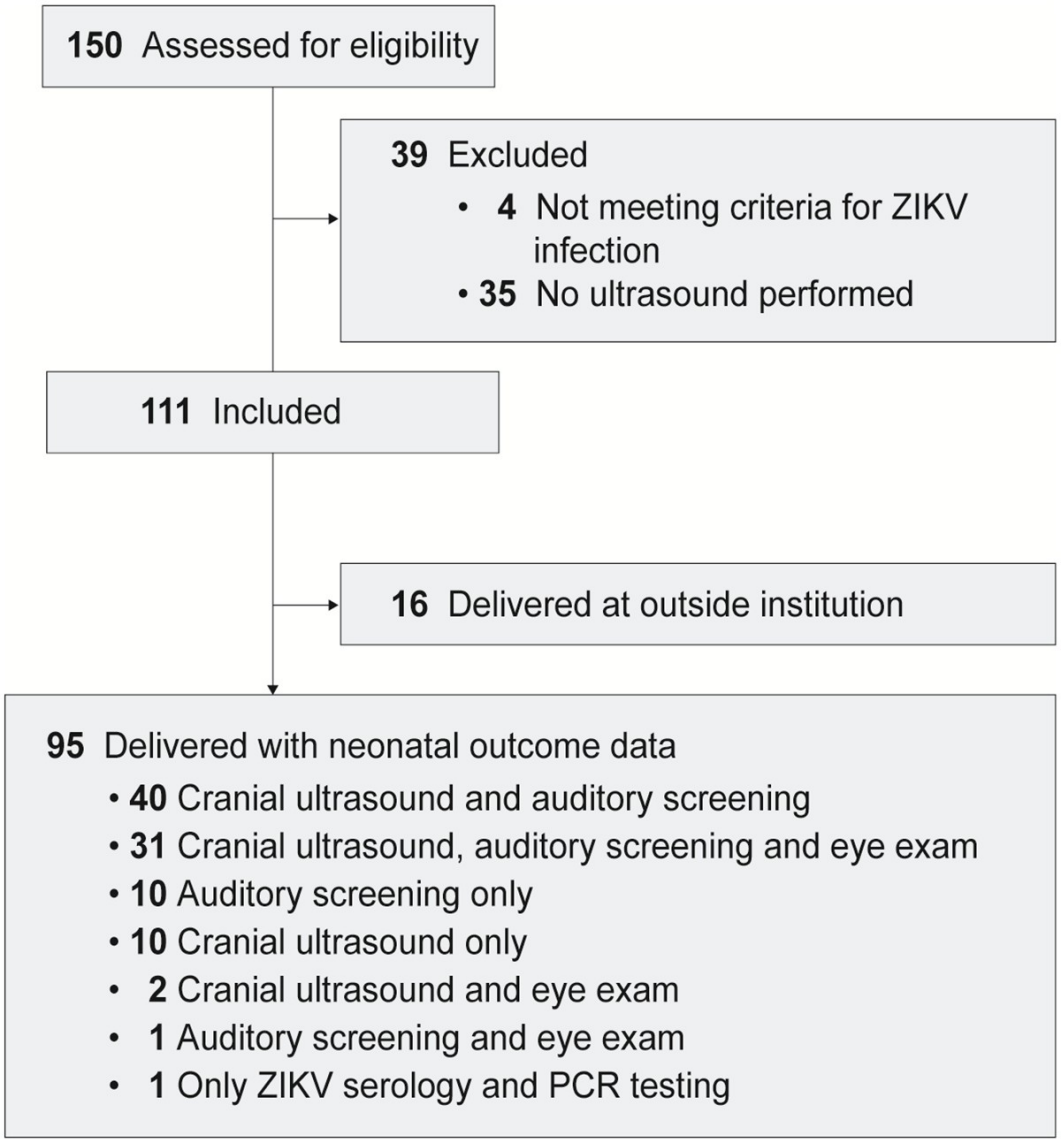
STROBE diagram to illustrate included and excluded subjects with distribution of postnatal testing in the neonates. Not all patients obtained the complete panel of recommended neonatal tests, which was a function of patient choice, cost of testing, pediatrician recommendation and limited knowledge of the spectrum of congenital injuries early in the study. Note that all neonates undergoing eye examination also had either a cranial ultrasound or auditory examination. Seven subjects also underwent MRI imaging of the fetal head. A single neonate only had postnatal serum ZIKV PCR and IgM, but did not have any imaging, auditory or vision testing.

Following delivery all neonates were evaluated by pediatricians for evidence of ZIKV-associated postnatal sequelae with initial ultrasound head imaging followed by MRI if abnormalities were seen or suspected. Serum and urine ZIKV testing was performed as detailed above. The pediatrician determined postnatal testing, which most often included a cranial ultrasound, auditory screening (automated auditory brainstem response) and serum testing (Fig 1). Additional studies including eye examination, MRI and cerebrospinal fluid (CSF) testing were completed due to provider discretion and findings of prior testing.

### Primary and secondary outcomes

The primary outcome was the diagnostic accuracy of a fetal body ratio (HC: FL), measured using prenatal ultrasound, to identify neonates with ZIKV-associated anomalies and abnormal postnatal testing. We evaluated two diagnostic thresholds for the HC: FL fetal body ratio: 1) the 10^th^ centile (Z-score ≤ -1 ·3) and, 2) the 3^rd^ centile (Z-score ≤ -2). A secondary outcome was the diagnostic accuracy of the HC: FL to detect ZIKV-associated anomalies and abnormal postnatal testing in normocephalic fetuses (HC > -2).

### Statistical analysis

Biometric measures from each ultrasound were recorded in millimeters (mm) with standardized Z-scores calculated based on gestational age at time of ultrasound. The performance of each measure to identify postnatal ZIKV infection was evaluated. Sensitivity, specificity, positive and negative predictive values (PPV and NPV, respectively), and positive and negative likelihood ratios (LR) were calculated. A true positive case had confirmed anomalies on postnatal evaluation that could be attributed to congenital ZIKV infection based on clinical exam, imaging, auditory and visual testing. Other fetal abnormalities not attributed to ZIKV infection are shown in S1 Table. True negatives were infants with no postnatal findings of congenital ZIKV infection. R studio (open source) software was used to perform statistical analysis.

## Results

### Patient characteristics

A total of 111 pregnant women were diagnosed with ZIKV infection and were included in this analysis (Fig 1). Acquisition of ZIKV occurred from local mosquito-borne transmission in Florida, sexual exposure or travel to endemic areas. The majority of women were asymptomatic (95/111) at the time of diagnosis and tested as part of a universal screening policy. Sixteen women were symptomatic, most commonly with a rash (14/16, 88%), fever (9/16, 56%), myalgia (5/16, 31%) and conjunctivitis (4/16, 25%) (Table 1, S2 Table). Using CDC diagnostic criteria, 6% of the women had an acute ZIKV infection (7/111, positive serum ZIKV PCR), 13% had ZIKV infection with unknown timing of infection and 81% had a probable ZIKV infection (90/111; S2 Table).

**Table 1:** Pregnancy, delivery and neonatal outcomes of pregnant women diagnosed with ZIKV infection.

Prenatal Demographics	All Subjects (N = 111)	Missing Postnatal Data (N = 17)	Normal Postnatal Findings^§^ (N = 73)	Any Abnormal Postnatal Finding^§^ (N = 24)	P value
Exposure trimester					
Preconception	2	0 (0)	1 (1)	0 (0)	NS
First	13	1 (6)	8 (11)	4 (17)	NS
Second	13	2 (12)	7 (10)	4 (17)	NS
Third	5	1 (6)	4 (5)	0 (0)	NS
Unknown	78	9 (53)	53 (73)	16 (67)	NS
Zika Symptoms^†^					
Fever	9	0 (0)	4 (5)	5 (21)	0.02*
Rash	15	0 (0)	10 (14)	5 (21)	NS
Myalgias	5	0 (0)	2 (3)	3 (13)	NS
Conjunctivitis	4	1 (6)	1 (1)	2 (8)	NS
Asymptomatic	95	15 (88)	62 (85)	18 (75)	NS
Gestational age at intake (weeks)	29.8	23.6 (7·9)	27.5 (7·7)	27.0 (6·2)	NS
Gestational age at delivery (weeks)	37.4	Unknown	38.3 (2·2)	39.0 (1·2)	NS
Birthweight (grams)	2810	Unknown	3052 (580)	3243 (452)	NS
Neonatal HC at birth (inches)	33.3	Unknown	33.4 (2·0)	34.0 (2.1)	NS
Neonatal HC Z-score at birth	0.7 (1.2)	Unknown	0.2 (1·0)	0.04 (1.7)	NS

Numbers shown are mean (standard deviation) or number (%).

HC, head circumference

P Values shown compare groups with normal and abnormal postnatal testing.

*P value <0.05. NS, not significant.

^†^Symptoms of possible ZIKV infection by subject recall to the obstetrical provider. Some subjects had more than one symptom, therefore percentages do not add up to 100.

^§^Postnatal findings considered to represent abnormalities possibly associated with ZIKV infection included findings consistent with congenital ZIKV infection as defined by the U.S. Centers for Disease Control and Prevention. [26]

### Pregnancy and neonatal outcomes

Of the 111 pregnancies, 16 women delivered at outside institutions; therefore, no birth outcomes were available. Of the 95 pregnancies with birth outcomes available, 14% (13/95) resulted in a preterm delivery ranging from 28 to 36 weeks with the remainder delivering at term with a mean gestational age of 39.7 ± 1.1 weeks (Table 1, S2 Table). Among preterm neonates, the mean birth weight was 2284.6 ± 661.8 grams (Z-score -0.2, adjusted for gestational age at delivery) with mean head circumference of 31.3 cm (SD 1.6, range 22–35 cm, Z-score -0·4). Term neonates delivered at an average gestational age of 39.1 weeks with mean birthweight of 3221.9 ± 410.7 grams (Z-score -0.1) and head circumference of 33·9 cm (SD 1.5cm, range 22–37 cm, Z-score -0.05).

Following delivery, 83 infants underwent cranial ultrasound, of which 7 had additional testing with MRI to define abnormalities noted on initial ultrasound imaging (Table 2). Among these 83 infants, 24 had abnormal imaging (29%) including intraventricular hemorrhage, caudothalamic groove cysts, lenticulostriate vasculopathy, intracranial calcifications, ventriculomegaly, extra-axial fluid collections and microcephaly, and one case of severe microcephaly with collapsed skull vault. No abnormal ultrasound findings were noted in infants that were born preterm.

**Table 2:** Prenatal and postnatal diagnosis of congenital injuries associated with ZIKV infection.

Type of Anomaly	N (%)
***Prenatal Diagnosis***	**14 (13)**
Prenatal Microcephaly (HC Z-score ≤ -2)	6 (5)
IUGR (AC Z-score ≤ -1.3)	10 (9)
Agenesis of the corpus callosum	1 (1)
Ventriculomegaly	1 (1)
***Postnatal Diagnosis***	**24 (25)**
Postnatal Microcephaly (HC at birth Z-score ≤ -2)	5 (5)
Small for gestational age (birthweight Z-score <-1.3)	12 (13)
Ventriculomegaly	1 (1)
Caudothalmic groove cysts/vasculopathy	16 (17)
Agenesis of the corpus callosum	1 (1)
Retinal imaging abnormalities	5 (5)
Calcifications	3 (3)
Polymicrogyria	3 (3)

The numbers shown reflect either the N (%) of congenital ZIKV-associated injuries out of either 111 fetuses undergoing ultrasound for prenatal diagnosis or 95 neonates evaluated by postnatal testing. Note that we show individual fetal anomalies in aggregate, rather than by individual cases, to prevent possible identification of individual neonates in this cohort with an unusual combination of anomalies. Therefore, the number of anomalies in the prenatal and postnatal categories do not add up to the total number of fetuses (N = 14) or neonates (N = 24) identified with anomalies.

### Prenatal microcephaly and fetal growth abnormalities

**Fig 2:**
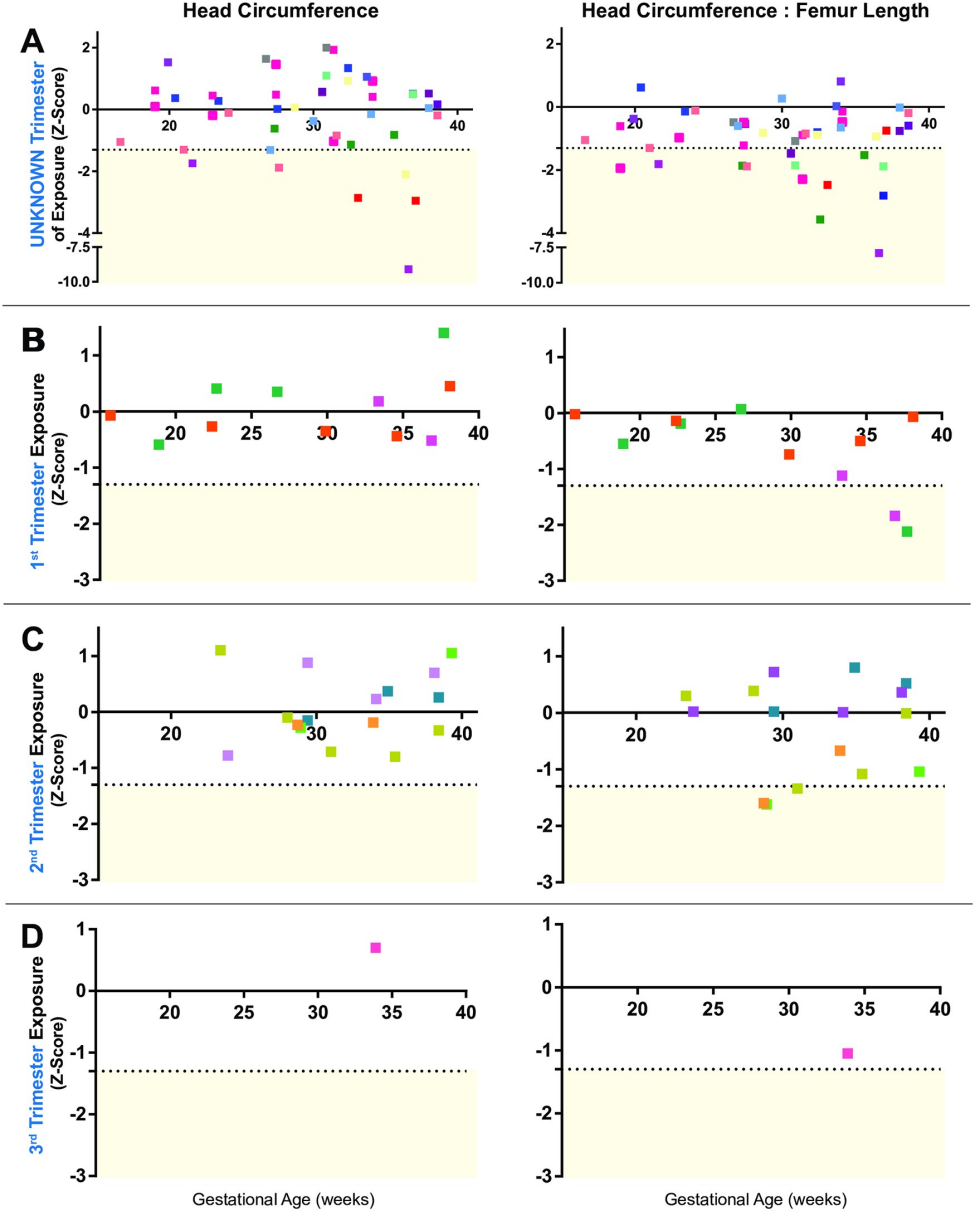
Head circumference and head circumference: Femur length ratio by trimester of ZIKV exposure. Z-scores for fetal head circumference (HC) and the HC to femur length (HC: FL) body ratio. The distribution of HC: FL is negatively skewed compared to HC measurements, indicating disproportionate growth with a smaller fetal head relative to the femur length. Each color represents data from a single ultrasound for an individual subject with colors kept consistent for each individual over time. The horizontal dotted line indicates a Z-score of -1.3, which corresponds to the 10^th^ centile. To simplify presentation of the data, data was shown separated by the best estimate for trimester of ZIKV exposure (A: unknown trimester, B: first trimester, C: second trimester, D: third trimester). Only the latest ultrasound per trimester for each subject was shown to allow discrimination between data points.

The mean gestational age at first prenatal ultrasound evaluation was 27.0 (±7·6) weeks. Over the course of pregnancy, each subject underwent an ultrasound approximately every 4 weeks after their initial visit, ranging from 14 to 40 weeks with an average of 2·4 (SD 1·4) ultrasounds per subject. Prenatal microcephaly, defined as an HC Z-score ≤ -2 (N = 6/111; Fig 2), was diagnosed in 5·4% of fetuses. IUGR was identified in 9% (10/111) of pregnancies based on AC Z-score ≤-1·3 (≤10^th^ centile; S3 Table). In contrast, 51% (56/111) of fetuses had a small HC: FL body ratio and 29% (N = 32/111) had an abnormal AC: FL body ratio at some time in pregnancy (Z-score ≤ -1·3, Table 3).

**Table 3:** Mean fetal biometric measures and body ratio Z-scores by gestational age strata.

Gestational Age Strata	HC	AC	FL	HC: FL	AC: FL	P values	
						FL vs. HC	FL vs. AC
All (N = 111)	0.2 (1.4)	0.5 (1·1)	0.9 (1.1)	-0.8 (1.2)	-0.4 (1.1)	<0.001*	<0.001*
>34 weeks (N = 81)	0.0 (1.7)	0.4 (1.2)	0.8 (1.4)	-0.8 (1.3)	-0.4 (1.2)	<0.001*	<0.001*
28–33 6/7 weeks (N = 70)	0.2 (1.0)	0.5 (1.2)	0.8 (1.8)	-0.6 (2.3)	-0.3 (1.6)	<0.001*	<0.001*
24–27 6/7 weeks (N = 37)	0.2 (1.2)	0.5 (0.9)	0.9 (1.0)	-0.7 (0.8)	-0.4 (0.8)	<0.001*	<0.001*
18–23 6/7 weeks (N = 39)	0.1 (1.0)	0.6 (1.0)	0.9 (1.0)	-0.7 (0.8)	-0.4 (0.7)	<0.001*	0.07

Mean Z-scores (standard deviation) are presented using the last ultrasound from each gestational age strata and estimated by INTERGROWTH-21^st^ standards. [28]

*P<0.05

HC, head circumference; AC, abdominal circumference; FL, femur length; HC: FL, head circumference to femur length ratio; AC: FL, abdominal circumference to femur length ratio.

### Diagnostic accuracy of HC: FL for prediction of abnormal postnatal testing

**Fig 3:**
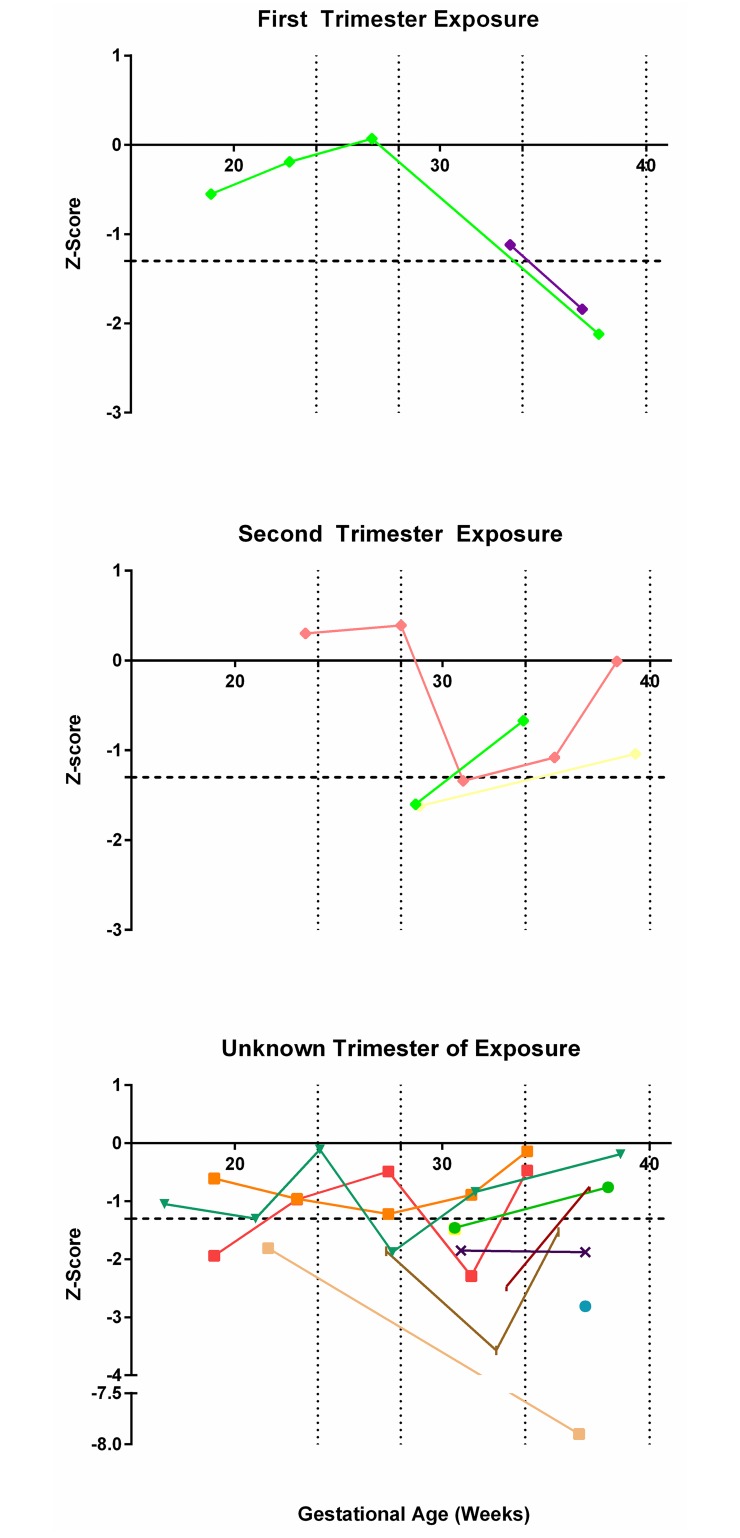
Head circumference: Femur length ratio by trimester of ZIKV exposure among neonates with abnormal postnatal testing.

Of the 95 neonates with postnatal testing, 25% (24/95) had abnormal postnatal testing associated with congenital ZIKV infection (Table 2). Congenital ZIKV injuries were most likely to be detected by either cranial ultrasound (20/83, 24%) or MRI (3/7, 43%); as not all postnatal tests were performed in all subjects, this result may be subject to selection bias. In contrast, an abnormal neonatal eye exam or auditory test occurred in 5/34 (15%) and 2/82 (2%), respectively. Overall, the sensitivity and PPV of either the fetal HC or HC: FL was poor for detection of abnormal postnatal testing associated with ZIKV congenital injury (Table 4). The best prenatal indicator for congenital ZIKV-associated injury was the diagnosis of microcephaly, but this occurred in only 5% (6/111) of fetuses in our cohort [HC Z-score ≤ -2; specificity 97%, 95% CI 90–99; NPV 80%, 95% CI 77–82; positive LR 3·6, 95% CI 0·8–16·6]. In contrast, an HC: FL ratio less than the 10^th^ (Z-score ≤ -1·3) or the 3^rd^ centile (Z-score ≤ -2) occurred in at least one ultrasound scan in 51% and 21% of fetuses, respectively. The distribution of the HC: FL ratio by trimester of ZIKV exposure for each subject with abnormal postnatal testing is shown in Fig 3.

**Table 4:** Diagnostic test performance of prenatal ultrasound for the detection of ZIKV-associated anomalies.

	Sensitivity	Specificity	PPV	NPV	LR+	LR-	
	*All Subjects (N = 95)*						
HC Z-score ≤ -2	13 (3–32)	97 (90–99)	50 (18–82)	80 (77–82)	3.6 (0.8–16.6)		0.9 (0.8–1.1)
HC: FL Z-score ≤ -1.3	58 (37–78)	52 (41–63)	25 (18–33)	82 (73–88)	1.2 (0.8–1.8)		0.8 (0.5–1.3)
HC: FL Z-score ≤ -2	25 (10–47)	90 (81–95)	40 (21–63)	81 (77–85)	2.4 (1.0–6.1)		0.8 (0.7–1.1)
	*Fetuses with a Normal Head Size (N = 89)*						
HC: FL Z-scores ≤ -1.3	57 (34–78)	52 (41–63)	23(16–32)	83 (74–89)	1·2 (0.8–1.9)		0.8 (0.5–1.4)
HC: FL Z-scores ≤ -2	20 (4–48)	89 (81–95)	23 (9–49)	87 (84–90)	1.8 (0.6–5.8)		0.9 (0.7–1.2)

Numbers shown reflect percent sensitivity, specificity, positive and negative predictive values, as indicated. Cases with aberrant growth were diagnosed by prenatal ultrasound measurement of fetal biometry with application of the INTERGROWTH-21^st^ sonographic standard to determine Z-scores for either head circumference (HC), abdominal circumference (AC) or head circumference to femur length (HC: FL), as shown above. Data is shown for all neonates with known postnatal outcomes (N = 95) and then, specifically for normocephalic fetuses (N = 89). Microcephaly was defined as an HC Z-score ≤ -2.

PPV, positive predictive value (true positive/(true positives + false positives); NPV, negative predictive value (true negatives/(true negatives + false negatives); LR+, likelihood ratio for positive test results (sensitivity/1-specificity); LR-, likelihood ratio for negative test results (1-sensitivity)/specificity; accuracy (true positives + true negatives)/all subjects

Next, we asked whether disproportionate growth of the fetal head with respect to the femur, using the HC: FL ratio, might represent a useful prenatal diagnostic test to exclude ZIKV-associated congenital injuries diagnosed postnatally. When a threshold at the 10^th^ centile for the HC: FL ratio was applied, there was a 52% specificity (95% CI 41–63%), 82% NPV (95% CI 73–88%) and negative LR 0.8 (0.5–1.3) for postnatal abnormalities. Application of a more stringent threshold for HC: FL at the 3^rd^ centile (Z-score ≤ -2) resulted in a 90% specificity (95% CI 81–95%), 81% NPV (95% CI 77–85%) and negative LR 1.2 (0.8–1.8), improving the ability to detect and rule out ZIKV-associated injury. We performed a final analysis to apply the test to normocephalic fetuses, where there is the most clinical uncertainty as to whether a congenital ZIKV-associated birth injury has occurred. In normocephalic fetuses, an HC: FL ratio at or below the 3^rd^ centile (Z-score ≤ -2) demonstrated similar specificity (89%, 95% CI 81–95%) with a superior NPV (87%, 95% CI 84–90%). Overall, the HC: FL ratio performed best for its NPV to exclude ZIKV-associated congenital injuries in the normocephalic fetus.

## Discussion

### Principal findings

The principal study finding is that an HC: FL ratio greater than the 3^rd^ centile in a normocephalic fetus is associated with an 87% NPV for the postnatal detection of congenital ZIKV-associated injuries. Although the diagnosis of microcephaly (HC Z-score ≤ -2) had a high specificity (97%) and NPV (80%), only 25% of neonates with congenital injury were diagnosed with microcephaly antenatally. The remaining 75% of neonates with abnormal postnatal testing were normocephalic *in utero*. This is consistent with prior studies showing higher rates of postnatal abnormalities than cases of prenatal microcephaly. [7, 14] Overall, the value of the HC: FL ratio as a prenatal diagnostic test is in its ability to provide reassurance of a healthy neonate to pregnant women with ZIKV infection in the setting of a normocephalic, non-anomalous fetus.

Notably, the sensitivity and PPV of the HC: FL ratio was poor, which is in accordance with other studies of the limited sensitivity of ultrasound to detect the broader spectrum of congenital ZIKV-associated injuries. [7, 14, 16] Unfortunately, the addition of fetal MRI to prenatal ultrasound does not appear to increase sensitivity for detection of congenital ZIKV-associated injuries. [9] A study to determine the diagnostic accuracy of a diverse set of sonographic abnormalities (e.g. placentomegaly, oligohydramnios) in combination with known ZIKV-associated anomalies found an adjusted odds ratio of 27.2 (95% CI 2·5–296·6) for abnormal neonatal outcomes. [16] The real-world application of various sonographic findings for the prediction of ZIKV-associated postnatal outcomes becomes challenging, particularly when there is a single abnormal finding that might be atypical of ZIKV-associated anomalies (e.g. oligohydramnios). The same study also found that fetal Doppler measurements of the middle cerebral artery were associated with abnormal neonatal outcomes (adjusted odds ratio of 20·2, 95% CI 3·2–132·6) [16]; these measurements can be technically difficult to obtain by sonographers with limited training in low- and middle-income countries. In contrast, obtaining the HC: FL ratio only requires that the sonographer measure the HC and FL, which represents skills typically learned early in ultrasound training. The simplicity of the HC: FL ratio makes it widely accessible to sonographers and obstetrical providers in high- and low-resource settings.

### Clinical and research implications

Ultrasound remains the most useful and easily available prenatal tool to evaluate fetuses exposed to ZIKV *in utero*. This study identifies a novel tool that may aid obstetricians, radiologists and pediatricians to more accurately predict fetuses and newborns with a lower risk for the greater spectrum of ZIKV-associated congenital injury. The concept of a femur-sparing profile of fetal growth restriction is quite new, but may represent a valuable indicator of fetal injury for other neuroinvasive, teratogenic viruses. Prospective trials are necessary to validate this tool and determine appropriate reference ranges, which may be population-specific. The long-term follow up of children affected by the congenital Zika syndrome, particularly those with a less severe phenotype, is essential to understand the range of congenital ZIKV injuries and their implications for thorough and adequate prenatal counseling.

### Strengths and limitations

The main study strength was the application of a novel prenatal diagnostic test for congenital injury ZIKV-associated injury that can be readily applied to low- and middle-income countries where ZIKV has become endemic. A second strength is the availability of prenatal ultrasound and postnatal imaging and testing data for nearly all participants. Study limitations include the retrospective nature of the study at a single center in the U.S and lack of a matched control group. However, INTERGROWTH-21^st^ standards were used to estimate Z-scores, which were derived from more than 13,000 pregnancies. Information regarding the spectrum of congenital ZIKV-associated injuries accumulated over the course of our study; therefore, not all neonates underwent complete postnatal testing early in the study, but 89% had at least a cranial ultrasound. Incomplete postnatal testing may have contributed to underestimation of the number of cases with congenital injury, which would bias our results towards the null hypothesis. We did not have access to maternal age or ethnicity for the participants as this was not obtained in our secondary analysis, but an earlier study containing nearly 80% of the same subjects reported maternal age and a multi-ethnic cohort. [24] Ideally, ethnic and population-specific fetal growth standards for fetal body ratios would be applied to our data [27]; however, fetal body ratio nomograms are available from the INTERGROWTH-21^st^ sonographic standard, which was a multi-ethnic study of fetal growth (18 countries) and has been previously used to evaluate ZIKV-associated congenital microcephaly in Brazil. [33] We acknowledge that diagnosis of ZIKV is complicated by cross-reactivity with dengue virus and other flaviviruses. In some of the cases in this study, a ZIKV diagnosis was made by laboratory testing positive for an “unspecified flavivirus”, which is a current testing limitation and is also inherent to other ZIKV pregnancy cohorts. Finally, although we focused the hypothesis on detection of a femur-sparing (disproportionate) profile of growth restriction, we recognize that fetuses with a symmetric (proportionate) growth restriction are also likely at risk for congenital ZIKV injury.

## Conclusions

Congenital ZIKV infection can significantly alter fetal neurodevelopment and result in lifelong neurologic morbidity including seizures, blindness, hearing loss, developmental delay, and motor impairment. [4, 12, 14] Prenatal diagnosis is currently limited to the recognition of structural brain anomalies by ultrasound or, in some cases, fetal MRI. In pregnancies that lack sonographic anomalies, we have no reliable test to detect postnatal sequelae. We identify the first prenatal diagnostic strategy to predict approximately 9 out of 10 fetuses that are at very low risk for the early postnatal detection of congenital injuries. A disproportionately small fetal head with respect to the femur may reflect a partial loss of neuroprogenitor cells and a more subtle phenotype of ZIKV-associated injury. Although the HC: FL ratio may offer some reassurance, the knowledge of long-term outcomes and developmental problems in children exposed to ZIKV *in utero* remains immature. Further research to link child outcomes with sonographic data obtained during pregnancy will be important for learning how we can prenatally diagnose the spectrum of less obvious injuries associated with congenital ZIKV infection.

## Supporting information

S1 Table Prenatal and postnatal diagnosis of other abnormalities not associated with ZIKV infection. (DOCX)

S2 Table Laboratory evidence for possible maternal ZIKV infection. (DOCX)

S3 Table Distribution of neonatal laboratory and clinical test results by abnormal HC and HC: FL body ratio. (DOCX)
