# Supplementary material for: Ultrasound prediction of Zika virus-associated congenital injury using the profile of fetal growth
Source: PLoS One. 2020 May 13;15(5):e0233023. doi: 10.1371/journal.pone.0233023 (PMC7219748; doi:10.1371/journal.pone.0233023)
Supplement: S1 Table — (DOCX) [file pone.0233023.s001.docx]

## Table S1. Prenatal and Postnatal Diagnosis of Other Abnormalities Not Associated with ZIKV Infection

| **Prenatal Diagnosis** | **9 (8)** |
| --- | --- |
| Oligohydramnios | 1 (1) |
| Polyhydramnios | 3 (3) |
| Situs inversus | 1 (1) |
| Pyelectasis | 2 (2) |
| Persistent right umbilical vein | 1 (1) |
| Increased nuchal translucency | 1 (1) |
| **Postnatal Diagnosis** | **4 (4)** |
| Choroid plexus cyst** | 3(3) |
| Atrial septal defect and patent ductus arteriosis | 1 (1) |

The numbers shown reflect either the N (%) of fetal abnormalities not attributed to ZIKV infection out of either 111 fetuses undergoing ultrasound for prenatal diagnosis or 95 neonates evaluated by postnatal testing. These findings did not contribute to our analysis, but are presented to reflect a more comprehensive picture of the cohort.

**We note that a choroid plexus cyst can be a normal variant, but list its presence in three cases because it occurred in combination with other anomalies.
