# Supplementary material for: Ultrasound prediction of Zika virus-associated congenital injury using the profile of fetal growth
Source: PLoS One. 2020 May 13;15(5):e0233023. doi: 10.1371/journal.pone.0233023 (PMC7219748; doi:10.1371/journal.pone.0233023)
Supplement: S2 Table — (DOCX) [file pone.0233023.s002.docx]

## Table S2. Laboratory Evidence for Possible Maternal ZIKV Infection

| **Subject** | **Symptom** | **Serum PCR** | **Urine PCR** | **ZIKV IgM #1** | **ZIKV IgM #2** | **ZIKV PRNT** | **DENV PRNT** | **Infant Serum PCR** | **Infant Urine PCR** | **Infant IgM** |
| --- | --- | --- | --- | --- | --- | --- | --- | --- | --- | --- |
| *First Trimester Exposure* | | | | | | | | | | |
| 1 | None | Neg | Neg | Pos | - | - | - | - | Neg | Neg |
| 2 | None | Neg | Neg | Pos | - | Equiv | Neg | - | - | - |
| 3 | None | - | - | Equiv | Neg | - | - | - | - | - |
| 4 | Rash, fever, myalgias | Neg | - | Pos | - | Pos | Pos | Neg | Neg | Neg |
| 5 | None | Neg | Neg | Pos | - | Neg | Pos | Neg | Neg | Neg |
| 6 | Rash | Neg | Neg | Pos | - | Pos | Pos | - | - | Neg |
| 7 | None | Neg | Neg | Pos | - | Pos | Pos | - | - | - |
| 8 | None | - | - | Pos | Neg | - | - | - | - | - |
| 9 | None | - | - | Pos | - | Pos | Pos | Neg | Neg | Neg |
| 10 | None | - | - | Pos | - | Pos | Pos | - | - | - |
| 11 | None | Neg | Neg | Pos | - | Pos | Pos | - | - | - |

| **Subject** | **Symptom** | **Serum PCR** | **Urine PCR** | **ZIKV IgM #1** | **ZIKV IgM #2** | **ZIKV PRNT** | **DENV PRNT** | **Infant Serum PCR** | **Infant Urine PCR** | **Infant IgM** |
| --- | --- | --- | --- | --- | --- | --- | --- | --- | --- | --- |
| *Second Trimester Exposure* | | | | | | | | | | |
| 12 | Rash, myalgias | Pos | Pos | Pos | - | Pos | Pos | Neg | Neg | Neg |
| 13 | None | Pos | Neg | Pos | Pos | Pos | Pos | Neg | Neg | Neg |
| 14 | None | Neg | Neg | Pos | - | Pos | Pos | Neg | Neg | Neg |
| 15 | None | Neg | Neg | Pos | - | Pos | Pos | Neg | Neg | Neg |
| 16 | None | Neg | Neg | Pos | - | Pos | Pos | Neg | Neg | Neg |
| 17 | None | - | Neg | Pos | - | Pos | Pos | Neg | Neg | Neg |
| 18 | Other | Neg | Neg | Pos | - | Neg | Neg | Neg | - | Neg |
| 19 | Myalgias, fever | Equiv | - | Pos | - | Pos | Pos | Neg | Neg | Neg |
| 20 | Rash, fever | Neg | Pos | Neg | - | - | Neg | Neg | Neg | Neg |
| 21 | Rash | Pos | Pos | Pos | - | Pos | Pos | - | - | - |
| 22 | Rash, fever, conjunctivitis | Neg | - | Pos | - | Pos | Pos | - | Neg | - |
| 23 | Rash, myalgias, fever, conjunctivitis | Pos | Pos | Pos | Pos | - | Neg | - | Neg | - |
| 24 | None | - | - | Pos | Neg | - | - | - | Neg | Neg |
| 25 | None | Pos | Pos | Pos | - | - | Pos | Neg | Neg | Neg |

| **Subject** | **Symptom** | **Serum PCR** | **Urine PCR** | **ZIKV IgM #1** | **ZIKV IgM #2** | **ZIKV PRNT** | **DENV PRNT** | **Infant Serum PCR** | **Infant Urine PCR** | **Infant IgM** |
| --- | --- | --- | --- | --- | --- | --- | --- | --- | --- | --- |
| *Third Trimester Exposure* | | | | | | | | | | |
| 26 | None | Neg | Neg | Pos | - | Pos | Neg | - | - | - |
| 27 | None | Neg | - | Pos | - | - | Pos | Neg | - | Neg |
| 28 | None | Pos | Pos | Pos | Pos | - | Pos | Neg | - | Neg |
| 29 | None | Neg | Neg | Pos | - | Pos | Pos | - | - | - |
| 30 | None | Neg | - | Pos | - | - | Pos | Neg | Neg | Neg |
| *Preconception Exposure* | | | | | | | | | | |
| 31 | None | Neg | Neg | Pos | - | Pos | Pos | - | - | - |
| 32 | Rash, fever | Neg | Neg | Pos | - | Pos | Pos | Neg | - | - |
| *Unknown Trimester of Exposure* | | | | | | | | | | |
| 33 | None | - | - | Equiv | - | Pos | Pos | Neg | Neg | Neg |
| 34 | None | - | Neg | Pos | - | Pos | Neg | Neg | Neg | Neg |
| 35 | None | Neg | Neg | Pos | - | Pos | Pos | - | - | - |
| 36 | None | Neg | Neg | Pos | - | - | - | - | - | - |
| 37 | None | Neg | Neg | Pos | Neg | Pos | Pos | - | - | Neg |
| 38 | None | Neg | Neg | Pos | - | Pos | Pos | Neg | Neg | Neg |
| 39 | None | Neg | Neg | Equiv | - | Pos | Pos | Neg | Neg | Neg |
| **Subject** | **Symptom** | **Serum PCR** | **Urine PCR** | **ZIKV IgM #1** | **ZIKV IgM #2** | **ZIKV PRNT** | **DENV PRNT** | **Infant Serum PCR** | **Infant Urine PCR** | **Infant IgM** |
| 40 | None | - | - | Pos | - | Pos | Pos | Neg | Neg | - |
| 41 | None | Neg | Neg | Pos | - | Pos | Pos | - | Neg | - |
| 42 | None | Neg | Neg | Pos | - | Pos | Pos | - | Neg | Neg |
| 43 | None | Neg | Neg | Pos | - | Pos | Pos | Neg | - | Neg |
| 44 | None | Neg | Neg | Neg | Neg | - | Neg | Neg | Neg | Neg |
| 45 | None | Neg | Neg | Pos | Neg | - | Neg | - | - | - |
| 46 | None | Neg | Neg | Pos | Neg | - | - | Neg | Neg | Neg |
| 47 | None | Neg | Neg | Pos | - | Equiv | Pos | - | Neg | Neg |
| 48 | None | Neg | Neg | Pos | - | Pos | Pos | - | - | - |
| 49 | None | - | - | Pos | - | Pos | Pos | - | - | - |
| 50 | None | Neg | Neg | Pos | Neg | Pos | Pos | Neg | Neg | Neg |
| 51 | None | Neg | Neg | Pos | - | Pos | Pos | Neg | Neg | Neg |
| 52 | None | Neg | Neg | Pos | - | Pos | Pos | Neg | Neg | Neg |
| 53 | None | - | - | Pos | - | Pos | Pos | Neg | Neg | Neg |
| 54 | None | Neg | Neg | Pos | - | Pos | Neg | Neg | Neg | Neg |
| 55 | None | Neg | Neg | Pos | - | Pos | Pos | Neg | Neg | Neg |
| 56 | None | - | - | Pos | - | - | - | Neg | Neg | Neg |
| **Subject** | **Symptom** | **Serum PCR** | **Urine PCR** | **ZIKV IgM #1** | **ZIKV IgM #2** | **ZIKV PRNT** | **DENV PRNT** | **Infant Serum PCR** | **Infant Urine PCR** | **Infant IgM** |
| 57 | None | Neg | Neg | Equiv | - | Pos | Pos | - | - | - |
| 58 | None | - | Neg | Pos | - | - | - | Neg | - | - |
| 59 | None | - | - | Pos | - | - | Pos | Neg | - | Neg |
| 60 | None | Neg | - | Pos | - | - | Pos | Neg | Neg | Neg |
| 61 | None | Neg | - | Equiv | Neg | Equiv | Pos | Neg | Neg | Neg |
| 62 | None | Neg | Neg | Pos | - | - | Neg | - | - | - |
| 63 | Rash, fever | - | - | Neg | - | - | Pos | Neg | - | Pos |
| 64 | None | - | - | Pos | - | Pos | Pos | Neg | Neg | Neg |
| 65 | None | Neg | Neg | Pos | - | - | - | Neg | Neg | Neg |
| 66 | None | Neg | Neg | Neg | Pos | - | - | - | - | - |
| 67 | None | Neg | Neg | Pos | - | - | Pos | - | Neg | Neg |
| 68 | Rash | Neg | - | Pos | - | Pos | Pos | Neg | Neg | Neg |
| 69 | None | Neg | Neg | Pos | - | Pos | Pos | Neg | - | Neg |
| 70 | None | Neg | Neg | Pos | - | Pos | Pos | Neg | Neg | Neg |
| 71 | None | Neg | Neg | Pos | - | Pos | Neg | - | Neg | Neg |
| 72 | None | Neg | Neg | Pos | Neg | - | - | Neg | - | Neg |
| 73 | None | Neg | Neg | Pos | - | - | - | Neg | Neg | Neg |
| **Subject** | **Symptom** | **Serum PCR** | **Urine PCR** | **ZIKV IgM #1** | **ZIKV IgM #2** | **ZIKV PRNT** | **DENV PRNT** | **Infant Serum PCR** | **Infant Urine PCR** | **Infant IgM** |
| 74 | None | Neg | Neg | Pos | Neg | - | - | Neg | - | Neg |
| 75 | None | Neg | - | Neg | Neg | Pos | Pos | Neg | Neg | Neg |
| 76 | None | - | - | Pos | - | Pos | Pos | - | - | Neg |
| 77 | None | - | - | Pos | - | Pos | Pos | Neg | Neg | - |
| 78 | None | Neg | Neg | Pos | - | Pos | Pos | - | Neg | - |
| 79 | None | Pos | Neg | Pos | Pos | Pos | Pos | Neg | Neg | Neg |
| 80 | None | Neg | Neg | Pos | - | Pos | Pos | - | - | - |
| 81 | None | Neg | - | Pos | - | Pos | Pos | - | Neg | - |
| 82 | None | Neg | Neg | Pos | - | Pos | Neg | Neg | Neg | Neg |
| 83 | None | Neg | Neg | Neg | Pos | Pos | Pos | Neg | - | Neg |
| 84 | None | Neg | Neg | Pos | Neg | Pos | Pos | Neg | Neg | Neg |
| 85 | None | Neg | Neg | Pos | - | Pos | Neg | Neg | Neg | Pos |
| 86 | None | Neg | Neg | Pos | - | Pos | Pos | - | - | - |
| 87 | None | Neg | Neg | Pos | - | Pos | Pos | Neg | - | Neg |
| 88 | None | Neg | Neg | Pos | - | Pos | Pos | Neg | - | - |
| 89 | None | Neg | Neg | Pos | - | - | - | - | - | - |
| 90 | None | Neg | Neg | Pos | - | Pos | Pos | Neg | Neg | Neg |
| **Subject** | **Symptom** | **Serum PCR** | **Urine PCR** | **ZIKV IgM #1** | **ZIKV IgM #2** | **ZIKV PRNT** | **DENV PRNT** | **Infant Serum PCR** | **Infant Urine PCR** | **Infant IgM** |
| 91 | None | Neg | Neg | Pos | - | Pos | Pos | - | - | - |
| 92 | None | Neg | Neg | Pos | - | Pos | Pos | - | - | - |
| 93 | None | Neg | Neg | Pos | - | Pos | Pos | - | - | - |
| 94 | None | Neg | Neg | Pos | - | Pos | Pos | - | - | - |
| 95 | None | Neg | Neg | Pos | - | Pos | Pos | - | - | - |
| 96 | None | Neg | Neg | Pos | - | Pos | Pos | Neg | Neg | Neg |
| 97 | None | Neg | Neg | Pos | - | Pos | Neg | Neg | - | - |
| 98 | None | Neg | Neg | Pos | - | Pos | Pos | - | - | Neg |
| 99 | None | - | - | Pos | - | - | - | - | - | - |
| 100 | None | Neg | Neg | Pos | - | Pos | Pos | - | Neg | - |
| 101 | None | Neg | Neg | Pos | - | Pos | Pos | - | - | - |
| 102 | Rash | Neg | Neg | Pos | - | Pos | Pos | - | - | - |
| 103 | Rash | Neg | Neg | Pos | - | Pos | Neg | - | - | Neg |
| 104 | None | Neg | Neg | Neg | Pos | - | - | - | - | - |
| 105 | None | Neg | Neg | Neg | Pos | - | - | - | - | - |
| 106 | None | Neg | Neg | Pos | - | - | - | - | - | - |
| 107 | None | Neg | Neg | Pos | - | Pos | Pos | - | - | - |
| **Subject** | **Symptom** | **Serum PCR** | **Urine PCR** | **ZIKV IgM #1** | **ZIKV IgM #2** | **ZIKV PRNT** | **DENV PRNT** | **Infant Serum PCR** | **Infant Urine PCR** | **Infant IgM** |
| 108 | None | Neg | Neg | Pos | - | Pos | Neg | - | - | - |
| 109 | None | Neg | Neg | Pos | - | - | - | - | - | - |
| 110 | Rash, myalgias, fever, conjunctivitis | - | - | Pos | - | - | Pos | Neg | Neg | Pos |
| 111 | Rash, fever, conjunctivitis | Neg | Neg | Pos | Neg | Pos | Pos | Neg | Neg | - |

PCR, polymerase chain reaction; ZIKV, Zika virus; DENV, dengue virus; PRNT, plaque reduction neutralization test; IgM, immunoglobulin M; Pos, positive; Neg, negative; Equiv, equivocal test result.

*Refers to a positive test result for an “undifferentiated flavivirus.”

**Negative ZIKV IgM result, but a West Nile Virus (WNV) microsphere immunoassay positive. Serologic cross-reactivity is known to occur among related flaviviruses, like WNV and ZIKV.
