# Supplementary material for: Ultrasound prediction of Zika virus-associated congenital injury using the profile of fetal growth
Source: PLoS One. 2020 May 13;15(5):e0233023. doi: 10.1371/journal.pone.0233023 (PMC7219748; doi:10.1371/journal.pone.0233023)
Supplement: S3 Table — (DOCX) [file pone.0233023.s003.docx]

## Table S3. Distribution of Neonatal Laboratory and Clinical Test Results by Abnormal HC and HC:FL body ratio

| Postnatal Testing | Total | HC ≤ -2*  (N= 6) | HC:FL ≤ -1.3* (N=56) |
| --- | --- | --- | --- |
| Serum ZIKV IgM (N=62)  Normal  Abnormal | 60  2 | 5 (8)  0 (0) | 29 (48)  1 (50) |
| CSF ZIKV IgM (N=6)  Normal  Abnormal | 3  3 | 1 (33)  2 (66) | 2 (67)  3 (100) |
| Serum PCR (N=58)  Normal  Abnormal | 58  0 | 6 (10)  0 (0) | 28 (48)  0 (0) |
| Head Ultrasound (N=83)  Normal  Abnormal | 63  20 | 2 (3)  3 (15) | 27 (43)  10 (50) |
| Magnetic Resonance Imaging (N=7)  Normal  Abnormal | 4  3 | 2 (50)  2 (67) | 1 (25)  2 (67) |
| Auditory Screening (N=82)  Normal  Abnormal | 80  2 | 2 (3)  1 (50) | 41 (51)  1 (50) |
| Eye Exam (N=34)  Normal  Abnormal | 29  5 | 1 (3)  2 (40) | 16 (55)  3 (60) |

HC, head circumference; HC:FL, head circumference:femur length body ratio

*Z-scores estimated by application of the IG-21 sonographic standard.
